# Direction-dependent biomechanical effects of transverse process hooks versus pedicle screws at the uppermost instrumented vertebra in adult spinal deformity: a finite element analysis

**DOI:** 10.3389/fbioe.2026.1937182

**Published:** 2026-09-25

**Authors:** Sun-Joon Yoo, Bong-Ju Moon, Jung-Do Eom, Young-Hoon Won, Jae Keun Oh, Kyung-Hyun Kim

**Affiliations:** 1 Department of Neurosurgery, Inha University College of Medicine, Incheon, Republic of Korea; 2 Department of Neurosurgery, Spine and Spinal Cord Institute, Gangnam Severance Hospital, Yonsei University College of Medicine, Seoul, Republic of Korea; 3 RAIDAM Co. Ltd., Wonju, Republic of Korea; 4 CGBio Co. Ltd., Seoul, Republic of Korea; 5 Department of Neurosurgery, Kangbuk Samsung Hospital, Sungkyunkwan University School of Medicine, Seoul, Republic of Korea

**Keywords:** adult spinal deformity, finite element analysis, pedicle screw, transverse process hook, uppermost instrumented vertebra, proximal junctional kyphosis, soft landing technique

## Abstract

Proximal junctional complications are often driven by abrupt mechanical transition at the uppermost instrumented vertebra (UIV). This study used patient-specific finite element analysis to compare biomechanical effects of transverse process hooks and pedicle screws at the UIV, specifically examining how hook orientation influences anterior-posterior load sharing. A validated nonlinear three-dimensional T7–L2 spinal model was developed using data from a 60-year-old woman. Three UIV constructs were evaluated at T9: bilateral pedicle screws (BPS) and downward and upward facing transverse process hook (TPH) configurations. Under a 500-N follower load and 8-Nm bending moments, we analyzed facet joint load (FJL), range of motion (ROM), intradiscal pressure, and mechanical failure risk. In flexion, FJL at the UIV+1/UIV level decreased by 6.2% and 19.6% in the downward-facing and upward-facing TPH models, respectively, compared with the BPS model. In extension, FJL decreased by 4.2% in the upward-facing TPH model, but increased by 32.4% in the downward-facing TPH model, relative to the BPS model. Both TPH models preserved greater UIV ROM than the bilateral pedicle screw model, with the upward-facing configuration achieving the highest flexion ROM (2.1°) and the downward-facing configuration achieving the highest extension ROM (3.2°). In the BPS model, UIV screw pull-out risk was 136% in flexion. Conversely, the risks of transverse process breakage for the downward-facing and upward-facing TPH models were substantially lower (50% and 42%, respectively). Hook constructs increased screw pull-out risk at the UIV-1 level compared with the BPS model. Using the TPH at the UIV can facilitate a biomechanical “soft landing” by preserving segmental motion and reducing posterior load at adjacent levels. These effects are orientation-dependent, with the upward-facing TPH configuration potentially offering greater advantage in preserving segmental motion during flexion. Surgical strategies should be tailored to individual bone quality due to potential stress transfer to the UIV-1 level.

## 1 Introduction

Long-segment spinal fusion is a standard surgical option for adult spinal deformity (ASD) to restore sagittal alignment and relieve symptoms. Despite its clinical benefits, the abrupt stiffness transition between the fused spine and the mobile segments often leads to proximal junctional kyphosis (PJK) and proximal junctional failure (PJF). Specifically, the concentration of excessive mechanical stress at the level of the uppermost instrumented vertebra (UIV) is a primary factor contributing to instrument failure including screw loosening and UIV fracture, which often necessitate revision surgeries (Nevzati et al., 2022).

The soft-landing technique aims to achieve a smooth transition in stiffness at the cranial end of the construct to prevent these mechanical problems. The use of a transverse process hook (TPH) rather than a pedicle screw has emerged as a promising biomechanical strategy that maintains motion at the adjacent levels and reduces the risk of PJK and PJF. Furthermore, clinical studies have demonstrated that TPHs not only reduce mechanical problems but also effectively prevent delayed-onset neurologic deficits by decreasing the risk of spinal cord compression (Lee et al., 2024; Yoo et al., 2024; Park et al., 2025).

While clinical evidence supports the efficacy of TPHs, the specific biomechanical mechanisms by which they distribute load across the transition zone, unlike rigid screws, remain incompletely understood. Previous finite element analysis studies have investigated various UIV strategies, including the use of tethers (Bess et al., 2017), long screws (Oe et al., 2023), bicortical screw fixation (Wui et al., 2020), and stiffness comparisons between different anchors (Nevzati et al., 2022). However, most of these studies focused primarily on the range of motion or localized stress distribution near the pedicle screws. Quantitative analyses regarding the load sharing between anterior elements, such as the intervertebral disc and posterior elements, including the facet joints, have been largely insufficient. Importantly, research on how specific surgical configurations such as hook orientation, alter this biomechanical behavior during physiological movements, including flexion and extension remain limited.

Accordingly, this study aimed to analyze the biomechanical behavior associated with UIV surgical strategies by comparing TPHs and pedicle screws using finite element models derived from patient-specific radiographic and computed tomography (CT) scans. Furthermore, our model reconstructed the facet joint articulations, enabling a better representation of actual physiological conditions. Specifically, by modeling the facet joint articulation, we aimed to reflect the physiological load-sharing conditions and quantitatively evaluate the direction-dependent load sharing and kinematic behavior based on hook orientation. Ultimately, this analysis sought to provide a comprehensive biomechanical rationale for the protective effects of TPHs observed in clinical practice.

## 2 Materials and methods

### 2.1 Finite element model

A patient-specific, nonlinear three-dimensional FE model of a thoraco-lumbar spine (T7–L2) was designed using the finite element analysis software ABAQUS (ABAQUS Inc., Johnston, RI, USA) from a preoperative CT scan of a 60-year-old female patient diagnosed with ASD, who underwent T9–S1 deformity fixation. Spinal segments below L3 were excluded from the model, as long-segment deformity fixation renders these levels effectively rigid. This study was approved by the Institutional Review Board of Yongin Severance Hospital (IRB No. 9-2026-0047), and the requirement for written informed consent was waived because of the retrospective nature of the study. The cortical bone and vertebral endplates were modeled with a uniform thickness of 1.0 mm. To secure both numerical stability and anatomical accuracy, the vertebrae and discs were meshed using a combination of three-dimensional hexahedral and tetrahedral elements (Figure 1A). Hexahedral elements (C3D8/C3D8H) were selected for the nucleus pulposus, annulus fibrosus, facet cartilage, endplates, and rods to ensure precision in non-linear stress analysis. For more complex geometries of the cortical and cancellous bone, as well as the screws and hooks, we applied tetrahedral elements (C3D4). The mesh size was strictly maintained between 0.5 and 1.0 mm, which was consistent with established literature indicating that sub-millimeter resolution is essential for achieving high anatomical reproducibility in patient-specific spinal model (Sensale et al., 2021). To compensate for the limited resolution of conventional CT images, the facet joint geometry was reconstructed based on anatomical references. The articular cartilage was explicitly defined with a 1.0-mm thickness, and the joint spaces were set to match the actual anatomical gaps identified on the CT images to closely replicate actual physiological kinematics (Figure 1B).

**FIGURE 1:**
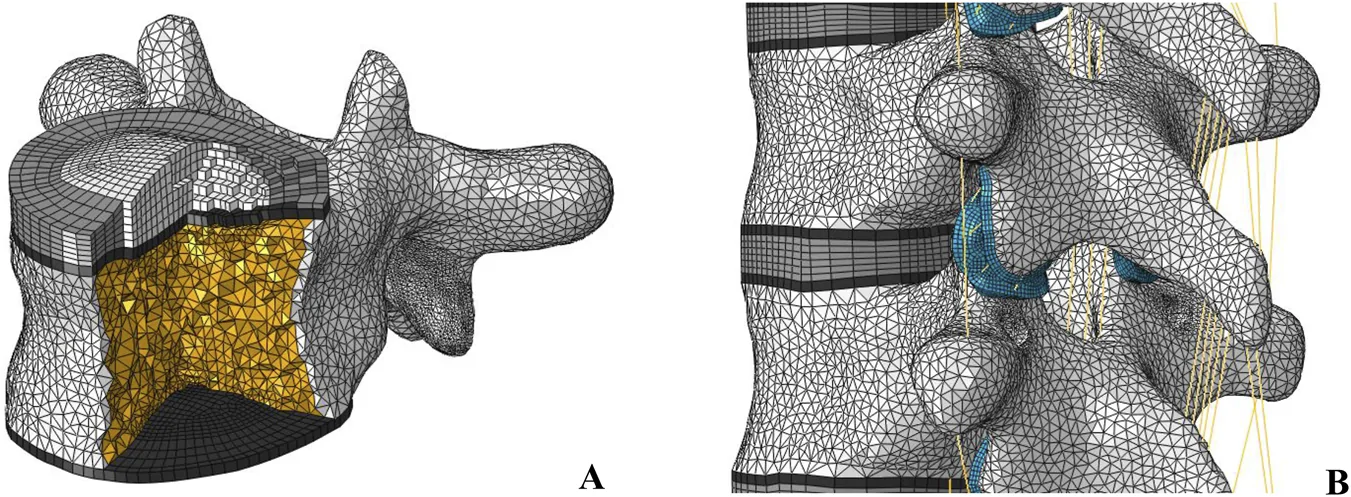
Patient-specific finite element model of the thoraco-lumbar spine. **(A)** Thoracic vertebral body of the finite element model (T8, cross-sectional view showing cortical bone, cancellous bone, and the intervertebral disc). **(B)** Detailed view of the thoraco-lumbar finite element model (facet joint cartilage highlighted in blue).

Based on previously published literature (Goel et al., 1995; Kopperdahl et al., 2002; Chen et al., 2009; Kabir et al., 2021), the material properties assigned to each component were assumed to be linear elastic, homogeneous, and isotropic (Table 1). The nucleus pulposus was modeled as an incompressible elastic solid with a Poisson’s ratio of 0.499 to simulate its fluid-like hydrostatic behavior. To represent the different tensile properties of soft tissues, spinal ligaments (anterior and posterior longitudinal ligaments, ligamentum flavum, interspinous ligament, supraspinous ligament, capsular ligament, and intertransverse ligament) and the annulus fibrosus were modeled as tension-only truss components using the “no compression” option (Ahn et al., 2008). The annulus fibrosus was represented by one-dimensional truss elements with a specific cross-sectional area, dispersed in a network pattern intersecting at 20° to 30° angles across the four layers of the annulus fibrosus. The instrumentation including the screws, hooks, and rods, was assigned the material properties of titanium alloy (Ti-6Al-4V; CDH Solera spinal system; Medtronic Sofamor-Danek, Memphis, TN, USA).

**TABLE 1:** Material properties in the finite element models.

Material	Elastic modulus (MPa)	Poisson’s ratio	Cross-sectional area (mm^2^)
Cortical bone	12,000	0.3	​
Cancellous bone	450	0.3	​
Bony end plate	500	0.4	​
Annulus ground	4.2	0.4	​
Nucleus pulposus	1	0.499 (incompressible)	​
Articular cartilage (facet joints)	10	0.45	​
Titanium (Ti-6Al-4V) (implant)	115,000	0.3	​
Annulus collagen fibers (AF)	358–550	​	0.24–0.5
Anterior longitudinal ligaments	7.8 (ε <12%)–20 (ε >12%)	​	49.1
Posterior longitudinal ligaments	10 (ε <11%)–20 (ε >11%)	​	22.2
Ligament flavum	15 (ε <6.2%)–19 (ε >6.2%)	​	71.7
Capsule ligaments	7.5 (ε <25%)–33 (ε >25%)	​	103.9
Intertransverse ligaments	10 (ε <18%)–59 (ε >18%)	​	1.8
Interspinous ligaments	10 (ε <14%)–12 (ε >14%)	​	49.2
Supraspinous ligaments	8 (ε <20%)–15 (ε >20%)	​	70.3

### 2.2 Surgical simulation

Three separate postoperative finite element models were created based on the T7–L2 spinal segment to study the biomechanical effects of different UIV anchoring methods. Under the guidance of an experienced spine surgeon, T12–L2 grade 1 osteotomies were performed. Additionally, pedicle screw insertion was modeled using a Boolean subtraction operation to ensure an accurate bone–screw thread interface. These simulated configurations were designed to directly reflect surgical techniques actively utilized in our clinical practice, with representative clinical cases provided to demonstrate the physical feasibility of each technique in actual ASD surgery (Figure 2).

**FIGURE 2:**
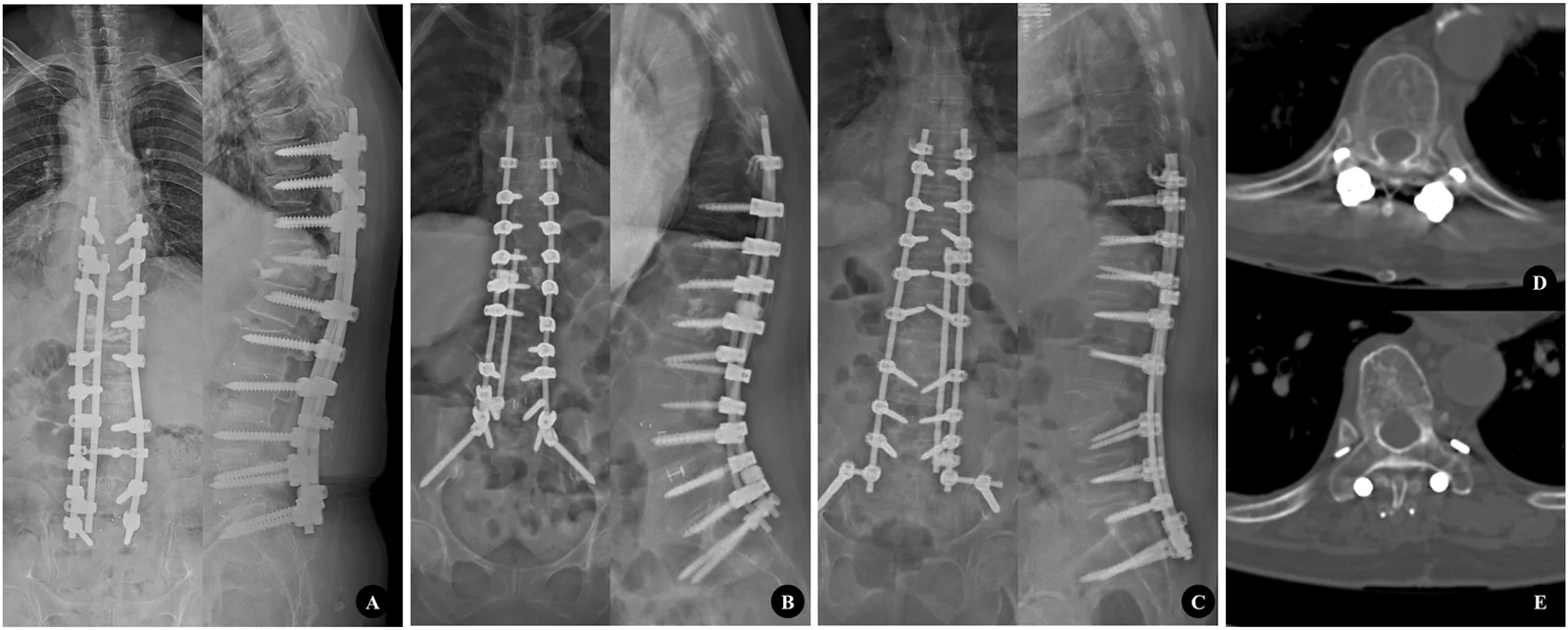
Representative clinical cases of the three uppermost instrumented vertebra (UIV) anchoring strategies at T9. **(A)** Radiographs of bilateral pedicle screw (BPS) fixation. **(B)** Radiographs of the downward-facing TPH construct. **(C)** Radiographs of the upward-facing TPH construct. **(D,E)** Axial CT images showing stable engagement of the downward-facing **(D)** and upward-facing **(E)** hooks.

The following three specific UIV constructs were modeled:

1. Bilateral pedicle screw (BPS) model (Control): A standard construct utilizing bilateral pedicle screws at the UIV (T9) and extending distally to L2 (Figure 3A)
2. Downward-facing TPH model: A hybrid construct utilizing transverse process hooks at the UIV (T9). The hooks were placed over the transverse processes in a downward orientation, with pedicle screws inserted from T10 to L2 (Figure 3B).
3. Upward-facing TPH model: A hybrid construct utilizing transverse process hooks at the UIV (T9). The hooks were placed beneath the transverse processes in an upward orientation, with pedicle screws inserted from T10 to L2 (Figure 3C).

**FIGURE 3:**
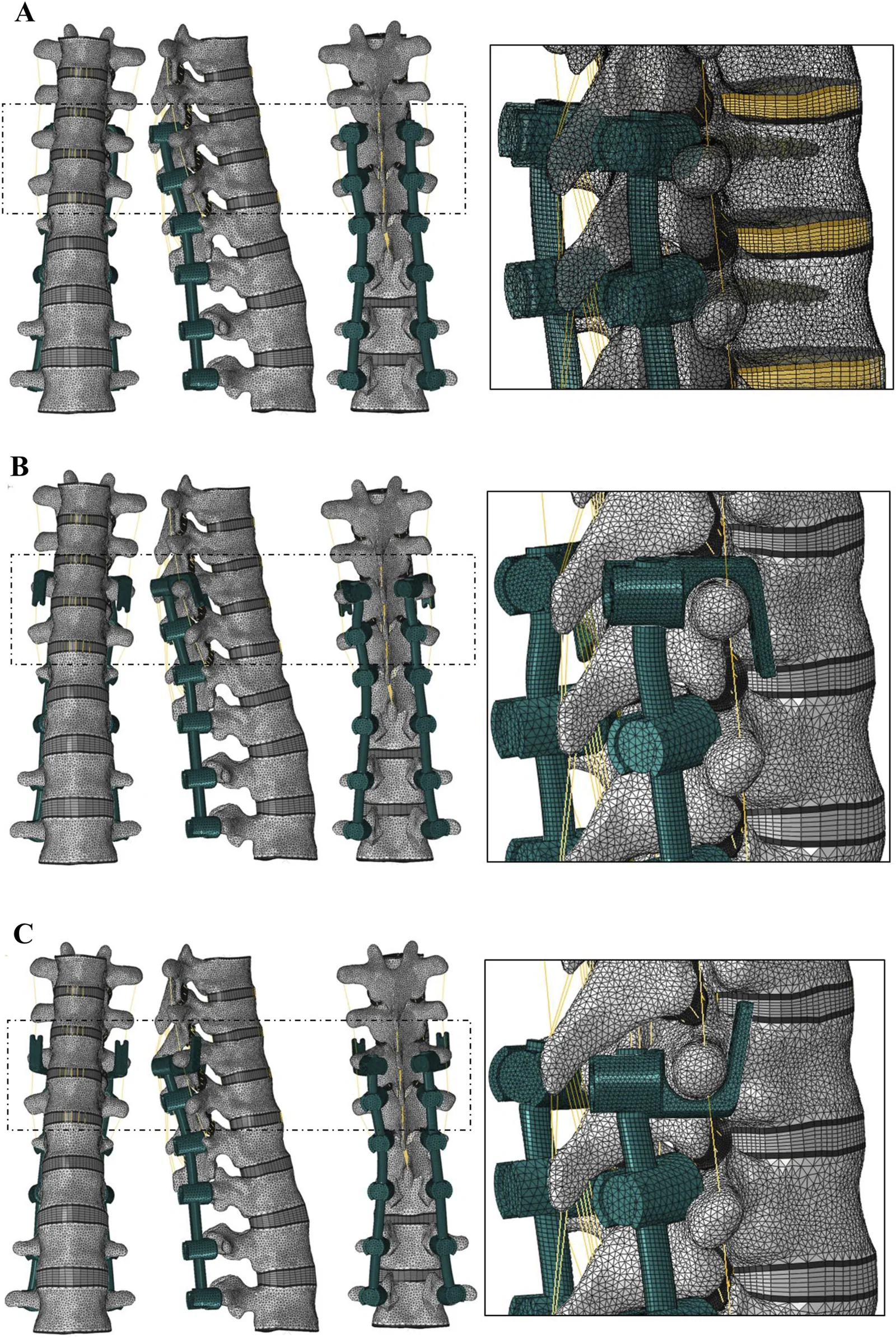
Postoperative finite element models illustrating the three uppermost instrumented vertebra (UIV) anchoring methods at T9. **(A)** BPS model (standard fixation with bilateral pedicle screws). **(B)** downward-facing TPH model (hybrid fixation with hooks positioned over the transverse processes). **(C)** upward-facing TPH model (hybrid fixation with hooks positioned beneath the transverse processes).

In all models, the implants were rigidly connected using 5.5-mm-diameter rods to simulate a fully assembled state.

### 2.3 Boundary and loading conditions

To simulate physiological loading and muscle stabilization, a 500-N follower load was initially applied along the spinal curvature. Because the modeled T7–L2 segment encompasses the thoraco-lumbar junction, this preload was selected to reflect physiological loading conditions of the lower thoracic and upper lumbar regions. Subsequently, a pure moment of 8 Nm was applied to the proximal spine to simulate physiological flexion and extension. This moment magnitude was selected to represent the transitional biomechanics between the thoracic (400N) (Sis et al., 2016) and lumbar spine (500–1200N) (Patwardhan et al., 2003; Rohlmann et al., 2009), which are typically evaluated at 5 Nm and 10 Nm, respectively. This intermediate loading protocol was adopted to reflect the transitional mechanical environment of the thoraco-lumbar junction, where the UIV of long-segment constructs is most commonly located. These consistent loading conditions were uniformly implemented across all experimental constructs, enabling consistent biomechanical comparisons among the different UIV strategies.

Furthermore, the distal end, specifically the inferior surface of L2, was entirely constrained in all degrees of freedom. This constraint was applied to simulate the actual patient’s rigid long-segment construct, which was fused down to the pelvis. This boundary condition effectively simulates the total restriction of the lumbar motion from long-segment fusion while preserving computational efficiency. In terms of contact conditions, facet joints were designed with frictionless finite-sliding contact to simulate normal joint movement. The bone-screw and screw-rod interfaces were assigned tie constraints to simulate rigid fixation. In contrast, the hook-transverse process interface was modeled as a frictionless finite-sliding surface-to-surface contact, allowing the hook to slide along and engage or separate from the transverse process (Figure 4).

**FIGURE 4:**
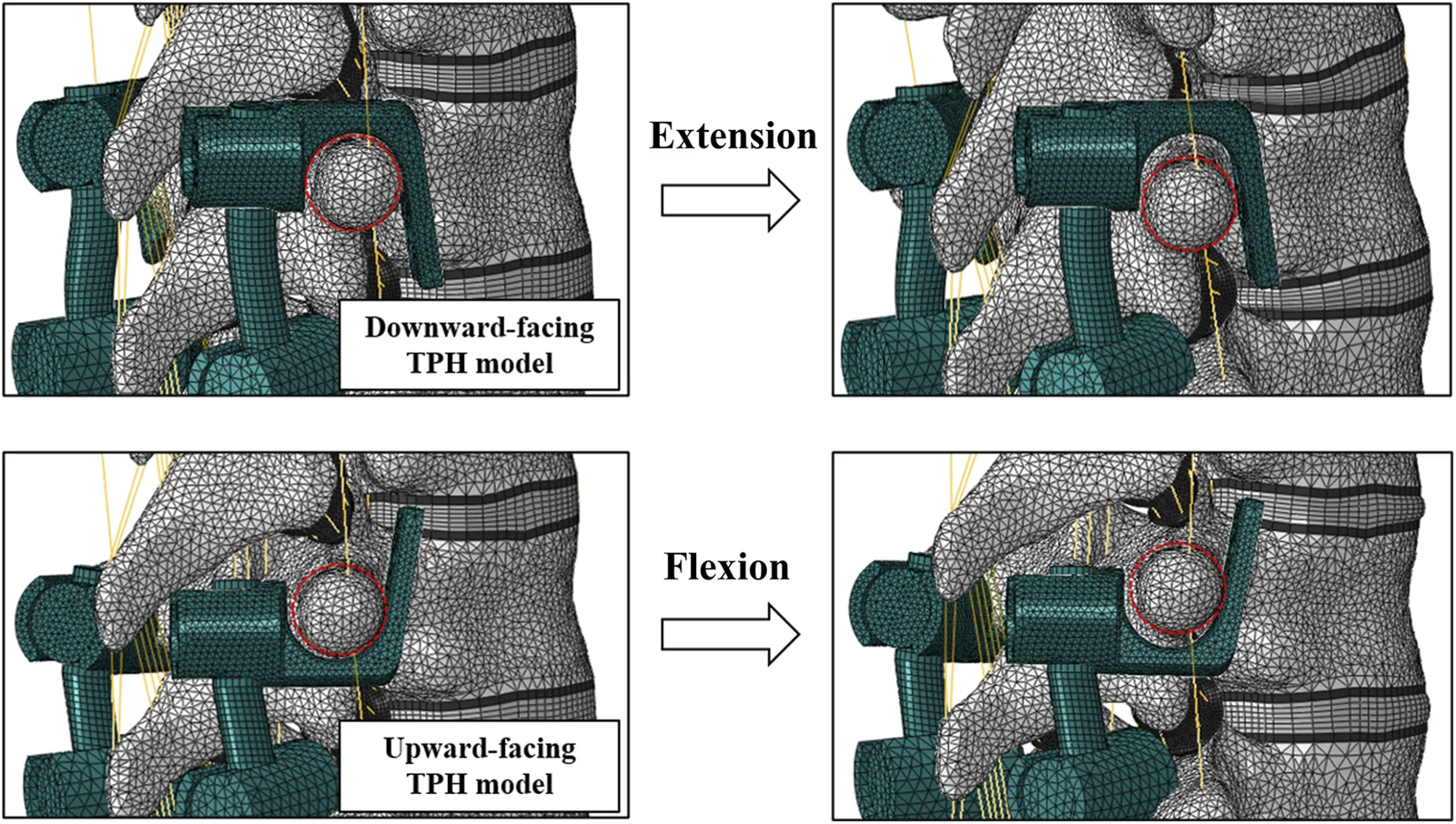
Direction-dependent hook–transverse process engagement behavior. The downward-facing TPH model loses structural engagement under extension loading, resulting in load redistribution to the disc and facet joint structures. In contrast, the upward-facing TPH model demonstrates gradual sliding engagement along the hook–transverse process interface under flexion loading, enabling simultaneous load sharing and motion preservation at the UIV. Red dashed circles indicate the transverse process location.

### 2.4 Data analysis

All simulations were executed using a dynamic implicit solver for quasi-static analysis. To quantitatively assess the biomechanical effects of the UIV instrumentation methods, we conducted kinematic and stress evaluations. The facet joint load (FJL) was calculated by averaging the normal contact forces across the left and right articular surfaces. The range of motion (ROM) was calculated by the change in Cobb angle during flexion and extension relative to the neutral posture under the initial 500-N follower load. Intradiscal pressure (IDP) was quantified by averaging the hydrostatic pressure at all elemental nodes within the nucleus pulposus.

Additionally, an assessment of mechanical failure risk was conducted using a relative failure risk index, defined as the ratio of peak von Mises stress (PVMS) to the yield stress of the specific bone type (Bayraktar et al., 2004). The study focused on three different failure scenarios: screw pull-out, evaluated using cancellous bone stress normalized to a 2 MPa yield stress (Kopperdahl and Keaveny, 1998); transverse process fracture, evaluated using cortical bone stress normalized to an 80 MPa yield stress (Bayraktar et al., 2004); and vertebral body fracture, also evaluated using cortical bone stress normalized to an 80-MPa yield stress limit.

### 2.5 Validation

To validate the physiological behavior of the model, predicted kinematic and loading responses were compared with postoperative dynamic radiographs of the index patient and previously published experimental data. The segmental ROM was measured from the postoperative standing dynamic (maximum flexion-extension) radiographs of the patient who underwent the downward-facing TPH construct, using the Cobb method between the superior and inferior endplates of the relevant vertebrae. The measured ROM showed trends consistent with those predicted by the downward-facing TPH model (Table 2). In the BPS model, segmental ROM under 8-Nm and 500-N compressive follower loading were 6.6° at UIV+1/UIV(T8–T9). This value was generally consistent with cadaveric studies reporting thoracic segmental ROM of approximately 4° under a 5-Nm moment after accounting for differences in loading magnitude (Wilke et al., 2025).

**TABLE 2:** Validation of the finite element model.

​	Segmental range of motion [°] (flexion to extension)	
Segments	Post-op dynamic x-ray images	FE model (downward-facing TPH)
UIV+1/UIV (T8-T9)	5.1	6.1
UIV/UIV-1 (T9-T10)	3.6	4.0
UIV-1/UIV-2 (T10-T11)	0.6	0.6

Regarding facet joint behavior, the model predicted FJLs (maximum 9.7 N) during flexion. Although facet loads during flexion are typically minimal *in vitro*, the addition of a 500-N follower preload likely contributed to the engagement of the facet articulations under physiological compression, consistent with previous cadaveric studies (Liu X. et al., 2018; Foroutan et al., 2024).

Although the rib cage was not included in the present model, previous *in vitro* studies have demonstrated that false ribs below T8 minimally affect thoracic ROM (Wilke et al., 2025). Therefore, the predicted motion at the UIV and adjacent levels is likely to remain physiologically reasonable. Furthermore, predicted IDPs ranged from 0.5 to 1.85 MPa, which falls within the range of previously reported *in vivo* lumbar measurements (0.5–2.0 MPa) (Wilke et al., 1999). Collectively, these validation results suggest that the model reasonably reproduces the biomechanical behavior of the thoracolumbar spine and provides a reasonable basis for relative comparisons among different UIV anchoring strategies under identical loading conditions.

## 3 Results

### 3.1 Facet joint load

In flexion, the BPS model exhibited an FJL of 9.7N at the UIV+1/UIV (T8–T9) level. In comparison, the downward-facing TPH and upward-facing TPH models showed reduced loads of 9.1N (−6.2%) and 7.8N (−19.6%), respectively (Figure 5A). In extension, the FJL at the UIV+1/UIV level was 3.6N in the BPS model. This load increased to 4.7N (+32.4%) in the downward-facing TPH model, but decreased to 3.4N (−4.2%) in the upward-facing TPH model. Meanwhile, at the UIV/UIV-1 level (T9–T10), only the downward-facing TPH model exhibited a measurable load of 16.6N, while negligible load was observed in the other models (Figure 5B).

**FIGURE 5:**
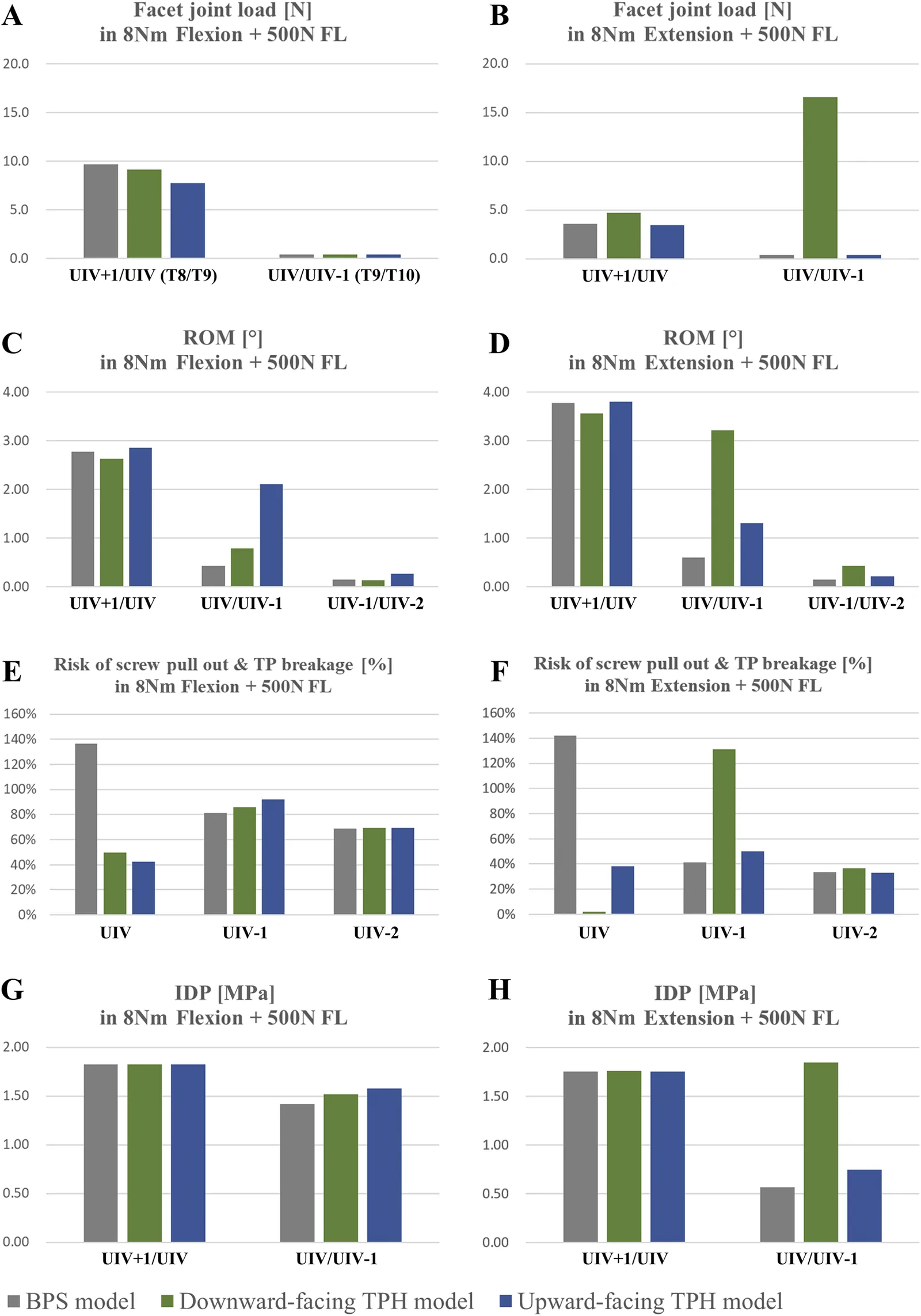
Biomechanical results of the three uppermost instrumented vertebra (UIV) constructs during flexion and extension. **(A,B)** Facet joint load (FJL). **(C,D)** Range of motion (ROM). **(E,F)** Risk of screw pull out and transverse process fracture. **(G,H)** Intradiscal pressure (IDP).

### 3.2 Range of motion

At the proximal adjacent segment (T8–T9), substantial differences in ROM were observed between models. However, at the UIV/UIV-1 level (T9–T10), the TPH models maintained a higher ROM than the BPS model. Specifically, the upward-facing TPH model exhibited the highest ROM of 2.1° in flexion (Figure 5C), and the downward-facing TPH model exhibited the highest ROM of 3.2° in extension (Figure 5D). These findings demonstrate greater preservation of UIV motion in the TPH models.

### 3.3 Risk of screw pull-out and transverse process breakage

The PVMS used to calculate the risk index was identified at the cancellous bone interface near the screw tip for the screw constructs (Figures 6A–C) and at the cortical bone interface of the transverse process for the hook constructs (Figures 6D,E).

**FIGURE 6:**
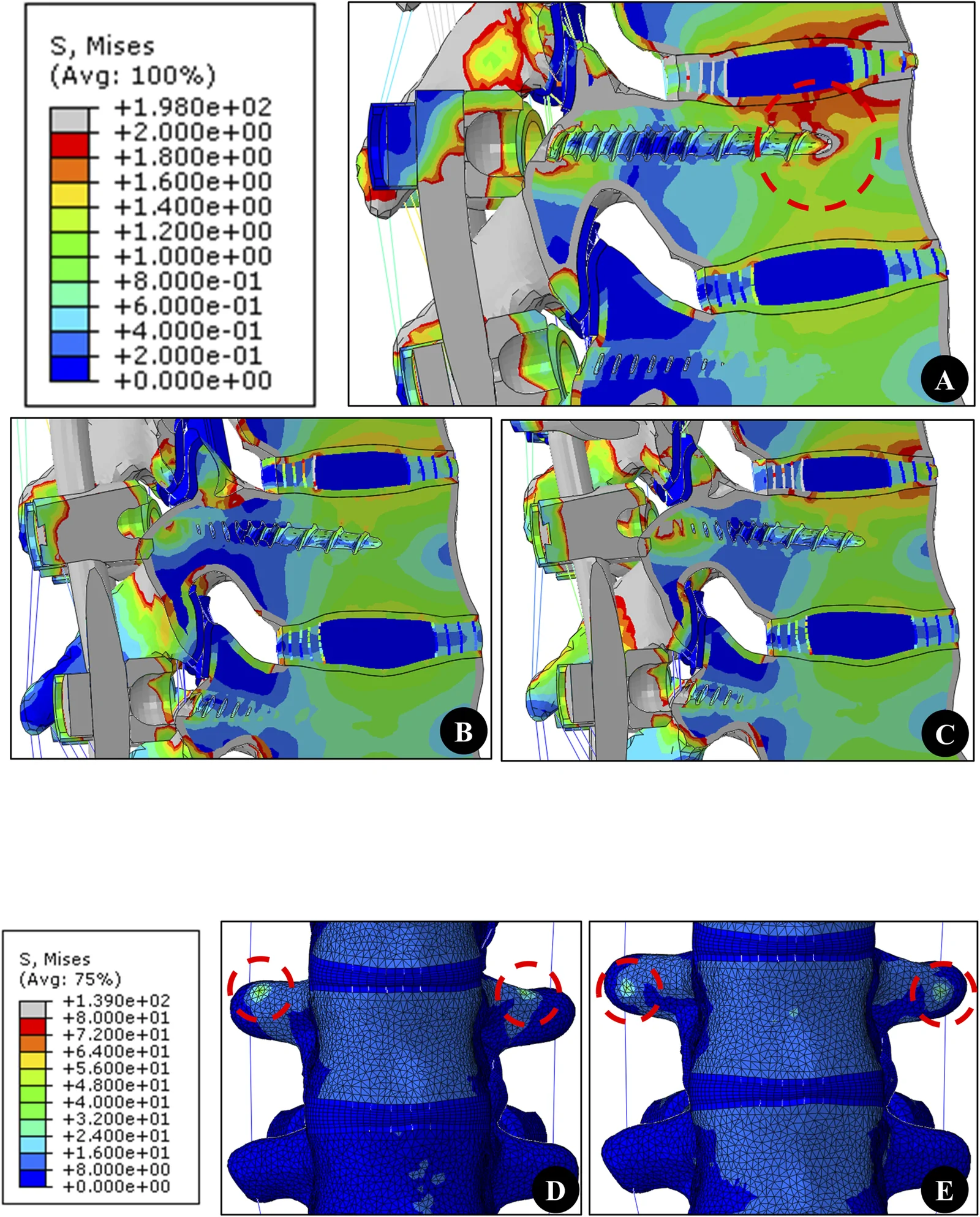
Peak von Mises stress (PVMS) distribution evaluating structural failure risk. **(A)** BPS model at T9 (UIV). **(B,C)** Pedicle screws at T10 (UIV-1) in the **(B)** downward-facing TPH and **(C)** upward-facing TPH models. **(D,E)** Transverse processes at T9 (UIV) in the **(D)** downward-facing TPH and **(E)** upward-facing TPH models. (Red dashed circles indicate primary stress concentrations).

In flexion, the BPS model exhibited a high risk index of 136% at the UIV level. In contrast, the TPH models exhibited lower risk indices at the UIV level, with the downward-facing TPH model recording 50% and the upward-facing TPH model 42% (Figure 5E). Nevertheless, at the UIV-1 (T10) level, the BPS model presented the lowest risk index at 81%, whereas the downward-facing TPH and upward-facing TPH models displayed higher risk levels of 86% and 92%, respectively.

In extension, the downward-facing TPH model exhibited minimal stress at the transverse process. However, at the UIV-1 level, the risk index for this model was 131%, similar to the 142% observed in the BPS model (Figure 5F).

### 3.4 Intradiscal pressure

In flexion, while proximal IDPs remained similar, the UIV/UIV-1 IDP increased by 7.0% and 11.3% in the downward-facing and upward-facing TPH models, respectively, than that of the BPS model (Figure 5G). Conversely, in extension, the UIV/UIV-1 IDP showed marked differences. The BPS and upward-facing TPH models demonstrated low pressures (0.57 MPa and 0.74 MPa), whereas the downward-facing TPH model showed an elevated IDP of 1.85 MPa, comparable to the highly loaded proximal levels (1.75–1.81 MPa; Figure 5H).

### 3.5 Risk of vertebral body failure

The risk of vertebral body failure was assessed by evaluating the PVMS on the anterior surface of the vertebral body during flexion. No substantial differences were observed across the models, with risk indices remaining consistent between 19% and 23% at the UIV and proximal adjacent levels.

## 4 Discussion

This study used a patient-specific finite element model to examine the impact of various anchoring procedures at the UIV on the biomechanical behavior of the UIV and adjacent segments in ASD surgery. Compared with traditional pedicle screws, TPHs demonstrated direction-dependent biomechanical characteristics, leading to distinct changes in ROM, FJL, IDP, and structural failure mechanisms. In the TPH models, FJL at the proximal adjacent level (UIV+1/UIV) decreased compared with the BPS model. Regarding structural failure, the BPS model was primarily susceptible at the UIV screw–bone interface, whereas the TPH models demonstrated a combined failure mechanism involving both the hook–transverse process interface at the UIV and the screw–bone interface at the UIV-1 level. Furthermore, the biomechanical effectiveness of TPHs varied with hook orientation and loading direction, indicating that their impact on the transition zone is direction-dependent.

In conventional long-segment spinal fusion for ASD surgery, UIV stiffness increases dramatically due to the rigid structure of the pedicle screws and connecting rods. This abrupt stiffness transition between the fixed UIV and the physiological mobility of the intact UIV+1 segment is widely recognized as a major mechanical contributor to PJK and PJF (Park et al., 2025). In this study, the BPS model showed substantially reduced ROM at the UIV/UIV-1 segment under applied bending moments, indicating excessively high bending stiffness. In contrast, the TPH models maintained a relatively high ROM at the UIV/UIV-1, exhibiting motion more comparable to the adjacent non-fixed spine. This indicates that certain hook configurations can maintain UIV motion within a physiological range, consistent with biomechanical studies demonstrating that TPHs effectively mitigate stiffness transfer effect (Thawrani et al., 2014; Metzger et al., 2016; Doodkorte et al., 2021; Nevzati et al., 2022). Despite this biomechanical basis, clinical results regarding the efficacy of TPHs are conflicting. Some studies have reported excellent PJK prevention with TPHs (Yoo et al., 2024), whereas others have reported PJK rates similar to or higher than those observed with screws (Matsumura et al., 2018; Tsutsui et al., 2022). The present analysis supports the concept of a “soft landing” with TPHs by demonstrating preservation of UIV motion, thereby providing a fundamental biomechanical basis for the ongoing clinical debate.

To evaluate anterior and posterior load-sharing mechanisms at the UIV and proximal adjacent segments, this study quantified IDP and FJL. Under flexion loading, the TPH models showed a distinct decrease in FJL at the UIV+1/UIV segment compared with the BPS model, accompanied by a slight increase in IDP at the UIV/UIV-1. This suggests that TPHs redistribute load, effectively reducing posterior mechanical stress on the adjacent segment while transferring some load to the anterior disc at the UIV/UIV-1. Because excessive facet joint loading is a primary contributor to facet hypertrophy, capsular damage, and subsequent adjacent segment degeneration, effectively reducing this posterior load with TPHs can play a major role in preventing facet-related clinical complications (Agarwal et al., 2018).

Clinically, proximal junctional problems are closely associated with structural failure, such as screw pull-out or compression fractures, rather than simple alignment changes. Moreover, insertion of a pedicle screw into the UIV inevitably carries the risk of superior articular facet violation, which may inadvertently impair posterior load sharing (Park et al., 2004). The present study revealed that applying a TPH shifted the primary structural failure mode from the cancellous screw–bone interface to the cortical hook–transverse process interface. Although pedicle screws generally provide superior fixation in healthy bone, their holding power depends largely on cancellous bone, which deteriorates rapidly with osteoporosis. Biomechanical studies indicated that in patients with osteoporosis, particularly those with volumetric bone mineral density around or below 100 mg/cm^3^ on quantitative CT, the cortical bone of the transverse process retains integrity better, making the fixation strength of TPHs comparable to that of pedicle screws (Bala et al., 2015; Ramchand and Seeman, 2018; Yang et al., 2024). Thus, TPHs may provide biomechanical advantages in preventing UIV structural failure in patients with osteoporotic ASD while preserving the facet joint. However, these benefits involve a biomechanical “trade-off” in the present study. Because TPHs distribute mechanical stress downward, they increased the risk of screw pull-out at the UIV-1 level compared with the BPS model. This trade-off was particularly pronounced in the downward-facing TPH model under extension loading, especially when the TPH loses engagement with the transverse process. In contrast, the upward-facing TPH model showed a slight increase in stress at the UIV-1 level compared with the BPS model, but this was minimal relative to the pronounced trade-off observed in the downward-facing TPH model. Moreover, in terms of the absolute pull-out risk index, the value predicted at the UIV-1 level in the upward-facing TPH model was markedly lower than that predicted at the UIV in the BPS model. Clinically, this suggests that the upward-facing TPH construct can produce a stress-distributing effect during both flexion and extension loading, potentially maintaining the stress level below the threshold for structural failure. Taken together, these findings indicate that TPH fixation does not simply relocate the failure risk to the UIV-1 level, but rather redistributes it in an orientation-dependent manner.

An important finding of this study was the direction-dependent biomechanical behavior of TPHs based on hook orientation. Crucially, these detailed observations were possible because the model in this study explicitly implemented facet joint articulation. Because facet joint spaces are notoriously difficult to distinguish on conventional CT scans, previous models have often omitted or oversimplified them. This study overcame these limitations by reconstructing the anatomical facet joint structure as closely as possible to the actual physiological state. This approach allowed us to quantify subtle changes in load distribution that were not captured by existing simplified models. Our findings suggest that the TPH is not simply a static fixed anchor but rather functions as a dynamic device that determines the “allowable direction of motion” and shares load depending on the loading conditions. Specifically, based on the hook’s orientation, it selectively permits motion in specific directions, which in turn regulates the load-transfer pathways and stiffness.

Under flexion load, the upward-facing TPH model mitigated excessive rotational stiffness by achieving structural engagement and load sharing, resulting in a reduction in FJL on adjacent segments while maintaining UIV/UIV-1 ROM. This suggests that the hook–transverse process interface in the upward-facing TPH model forms a slight incline, enabling partial load sharing at the UIV while allowing for motion.

In contrast, the downward-facing TPH model under extension loading showed increased UIV/UIV-1 ROM accompanied by elevated FJL at the UIV/UIV-1 level and UIV/UIV-1 IDP. This indicates that the downward-facing TPH construct provides insufficient support during extension, as structural engagement is lost. Consequently, the mechanical load is redistributed to the disc and facet joint structures, bypassing the hook. These findings highlight the importance of strategically determining hook orientation by considering the patient’s postoperative sagittal plane alignment, expected primary loading direction, and preservation of UIV segment motion (Figure 4).

Cadaver and *in vivo* studies evaluating the biomechanical effects of UIV fixation have fundamental limitations, including high specimen-to-specimen variability and structural damage during repeated experiments, and altered joint motion caused by intra-articular sensor insertion. Furthermore, direct quantification of bone–implant interface stress distribution prior to failure is experimentally unfeasible. This finite element analysis addressed these limitations by non-invasively examining load distribution under identical conditions. The results align with previous *in vitro* findings (Patwardhan et al., 2003; Du et al., 2016; Liu T. et al., 2018) showing that compressive preloads induce facet loading even during flexion. Therefore, the facet joint modeling in this study reasonably simulated the biomechanical response, supporting the validity of relative biomechanical comparisons among UIV anchoring strategies.

This study had several limitations. First, the finite element model was based on data from a single patient. Second, PVMS usage to assess failure risk does not directly predict actual clinical fractures. Third, the model did not include the rib cage. However, this study focused on a relative biomechanical comparison under identical conditions rather than absolute stress values. Previous studies indicate that false ribs at T8 and below have a minimal impact on segmental motion (Sis et al., 2016; Wilke et al., 2025). Therefore, excluding the rib cage is unlikely to alter the comparative biomechanical trends between the downward facing and upward facing hooks. Fourth, the radiographic ROM used for validation was measured in two dimensions with an inter-/intra-observer error on the order of 2°–3°, and precise landmark definition on projectional radiographs is inherently limited. Therefore, the comparison with the three-dimensional FE predictions was used only to confirm physiologically reasonable motion trends rather than for quantitative validation. Moreover, because finite element analysis relies on idealized assumptions regarding material properties, interface conditions, and loading, the present biomechanical findings should be interpreted with caution and confirmed through further clinical studies.

## 5 Conclusion

The findings of this study are meaningful. By explicitly modeling the facet joint articulations to reproduce the actual physiological state, this model successfully quantified subtle changes in mechanical load distribution. Furthermore, measuring the specific FJL for each implant type and evaluating the ROM through this explicit facet implementation has rarely been demonstrated in previous research. Replacing pedicle screws with a TPH at the UIV promotes a biomechanical “soft landing” by preserving segmental motion and reducing FJL at adjacent levels. Although the downward-facing TPH configuration is conventionally preferred, the upward-facing TPH approach could offer biomechanical advantages in optimizing load-sharing during flexion. Because the application of TPHs inevitably transfers some mechanical stress to the UIV/UIV-1 level, applying a uniform surgical strategy across all patients should be avoided. Achieving optimal surgical outcomes requires a tailored strategy that comprehensively considers the individual patient’s bone quality, expected loading demands, and the specific biomechanical characteristics of each hook orientation.

## Data Availability

The raw data supporting the conclusions of this article will be made available by the authors, without undue reservation.
