# Correction: Direction-dependent biomechanical effects of transverse process hooks versus pedicle screws at the uppermost instrumented vertebra in adult spinal deformity: a finite element analysis

**DOI:** 10.3389/fbioe.2026.2034046

**Published:** 2026-10-29

**Authors:** Sun-Joon Yoo, Bong-Ju Moon, Jung-Do Eom, Young-Hoon Won, Jae Keun Oh, Kyung-Hyun Kim

**Affiliations:** 1 Department of Neurosurgery, Inha University College of Medicine, Incheon, Republic of Korea; 2 Department of Neurosurgery, Spine and Spinal Cord Institute, Gangnam Severance Hospital, Yonsei University College of Medicine, Seoul, Republic of Korea; 3 RAIDAM Co. Ltd., Wonju, Republic of Korea; 4 CGBio Co. Ltd., Seoul, Republic of Korea; 5 Department of Neurosurgery, Kangbuk Samsung Hospital, Sungkyunkwan University School of Medicine, Seoul, Republic of Korea

**Keywords:** adult spinal deformity, finite element analysis, pedicle screw, transverse process hook, uppermost instrumented vertebra, proximal junctional kyphosis, soft landing technique

The funder “Yonsei University College of Medicine”, (6-2024-0185) was erroneously omitted. The correct **Funding** statement reads:

“The author(s) declared that financial support was received for this work and/or its publication. This study was supported by a faculty research grant of Yonsei University College of Medicine (6-2024-0185).”

The original version of this article has been updated.

